# 1-[2-(2-Methoxyphenylamino)ethylamino]-3-(naphthalene-1-yloxy)propan-2-ol May Be a Promising Anticancer Drug

**DOI:** 10.3390/molecules191221462

**Published:** 2014-12-19

**Authors:** Tomoyuki Nishizaki, Takeshi Kanno, Ayako Tsuchiya, Yoshiko Kaku, Tadashi Shimizu, Akito Tanaka

**Affiliations:** 1 Division of Bioinformation, Department of Physiology, Hyogo College of Medicine, 1-1 Mukogawa-cho, Nishinomiya 663-8501, Japan; E-Mails: kanno@hyo-med.ac.jp (T.K.); ayako-t@hyo-med.ac.jp (A.T.); yoshikokaku0506@gmail.com (Y.K.); 2 Laboratory of Chemical Biology, Advanced Medicinal Research Center, Hyogo University of Health Sciences, 1-3-6 Minatojima, Chuo-ku, Kobe 650-8530, Japan; E-Mails: furanether2007@yahoo.co.jp (T.S.); reprori2000@yahoo.co.jp (A.T.)

**Keywords:** HUHS1015, naftopidil analogue, anticancer drugs, necrosis, apoptosis

## Abstract

We have originally synthesized the naftopidil analogue 1-[2-(2-methoxyphenylamino)ethylamino]-3-(naphthalene-1-yloxy)propan-2-ol (HUHS 1015) as a new anticancer drug. HUHS1015 induces cell death in a wide variety of human cancer cell lines originated from malignant pleural mesothelioma, lung cancer, hepatoma, gastric cancer, colorectal cancer, bladder cancer, prostate cancer, and renal cancer. HUHS1015-induced cell death includes necrosis (necroptosis) and apoptosis, and the underlying mechanism differs depending upon cancer cell types. HUHS1015 effectively suppresses tumor growth in mice inoculated with NCI-H2052, MKN45, or CW2 cells, with a potential similar to or higher than that of currently used anticancer drugs. Here we show how HUHS1015 might offer brilliant hope for cancer therapy.

## 1. Introduction

Naftopidil, an antagonist for the α_1_-adrenoceptor, with high selectivity for α_1A_- and α_1D_-receptors, has been clinically used as a drug for treatment of benign prostate hyperplasia and hypertension [1]. Accumulating evidence has shown that naftopidil exhibits an anticancer effect. Naftopidil inhibits prostate cancer cell growth by arresting them at the G_1_ phase of cell cycling [2,3]. In our studies, naftopidil induced cell death for bladder, prostate, renal cancer, and malignant pleural mesothelioma (MPM) cell lines [4,5]. The mechanism for the anticancer action of naftopidil remains to be explored. α_1_-Adrenoceptor is divided into α_1A_-, α_1B_-, and α_1D_-subtypes, and the receptor is linked to G_q/11_ protein-bearing phospholipase C activation followed by protein kinase C (PKC) activation [6,7,8]. Therefore, one would speculate that naftopidil should suppress PKC activation by inhibiting the α_1_-adrenoceptor. Surprisingly, the PKC inhibitor GF109203X attenuated naftopidil-induced apoptosis of MPM cells [5]. Moreover, knocking down the α_1D_-adrenoceptor promoted proliferation of MPM cells [5]. Collectively, these data suggest that naftopidil induces MPM cell death by a mechanism independent of α_1_-adrenoceptor blocking.

**Figure 1:**
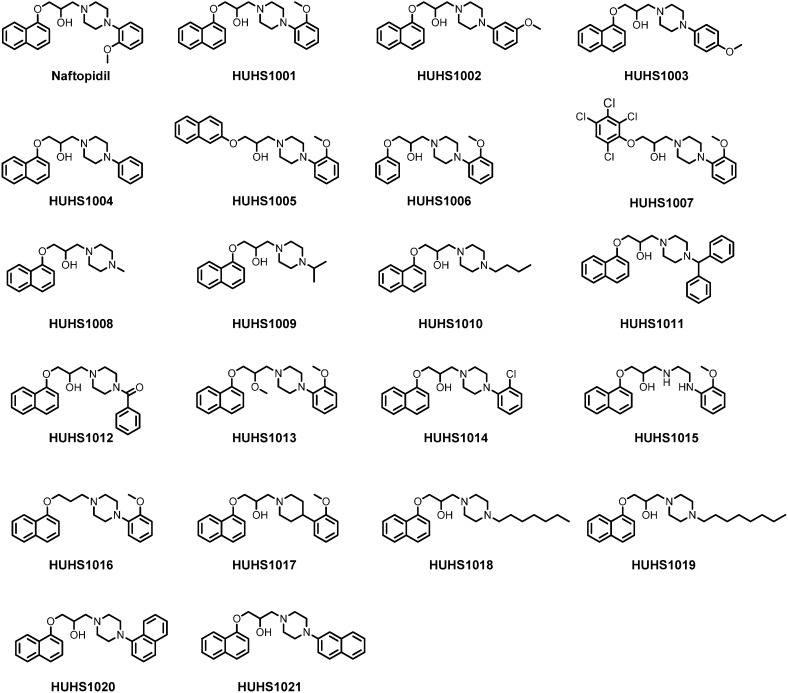
Chemical structures for naftopidil and newly synthesized naftopidil analogues.

We have synthesized 21 naftopidil analogues (Figure 1) and assessed the anticancer effect of each compound.

## 2. Synthesis of HUHS1015

2-[(2-Methoxyphenyl)aminoethyl]amine (200 mg, 1.2 mmol) was added to a solution of 2-[(1-naphthoxy)methyl]oxirane (482 mg, 2.4 mmol) in ethanol (3 mL) at room temperature, and the mixture was stirred for 1 h at reflux. The reaction mixture was concentrated *in vacuo*. The crude product was purified by silica gel column chromatography to give HUHS1015 (11 mg, 1.2%). ^1^H-NMR (400 MHz, CDCl_3_) d2.91–3.05 (m, 4H), 3.31 (t, *J* = 6.0 Hz, 2H), 3.82 (s, 3H), 4.13–4.26 (m, 3H), 6.64–6.71 (m, 2H), 6.77 (d, *J* = 7.8 Hz, 1H), 6.82 (d, *J* = 7.8 Hz, 1H), 6.87 (t, *J* = 7.8 Hz, 1H), 7.36 (dd, *J* = 8.2 and 7.8 Hz, 1H), 7.43–7.50 (m, 3H), 7.80 (d, *J* = 8.3 Hz, 1H), 8.23 (d, *J* = 7.8 Hz, 1H); ESI-HRMS (positive ion, sodium formate) calcd for C_22_H_27_N_2_O_3_ ([M+H^+^]): 367.2016; Found 367.2054.

## 3. The Naftopidil Analogue HUHS1015 Induces Cell Death for a Wide Variety of Human Cancer Cell Lines

**Figure 2:**
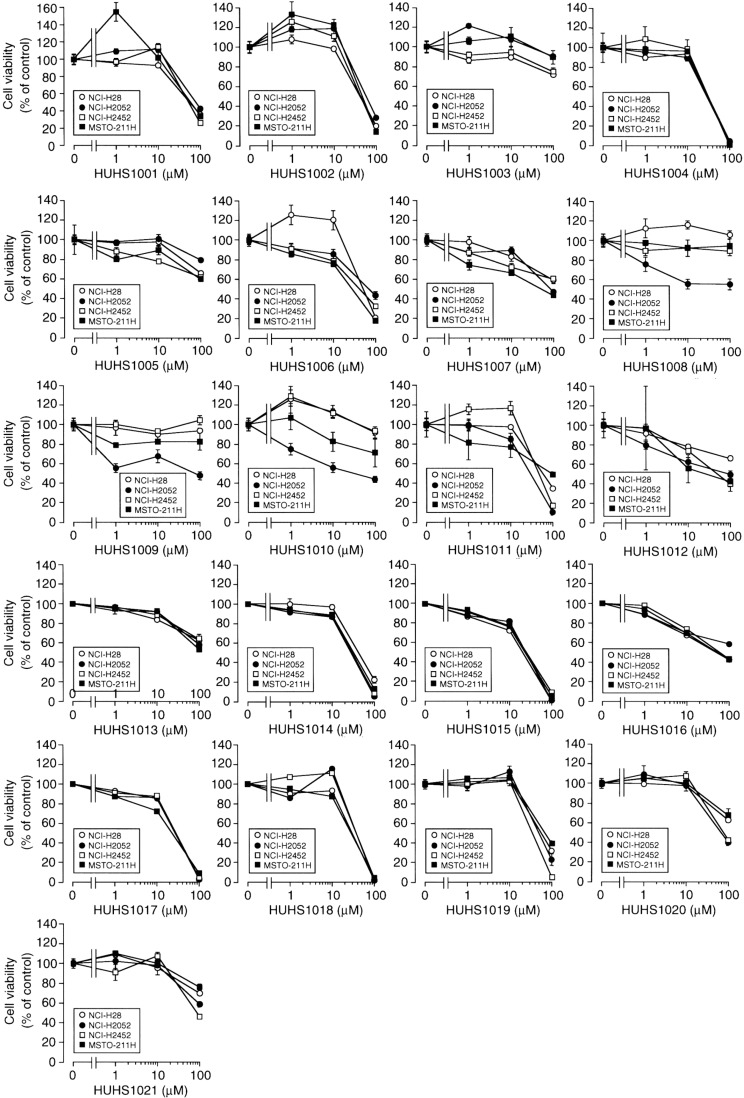
The effect of naftopidil analogues on cell viability for malignant mesothelioma cell lines. MTT assay was carried out in NCI-H28, NCI-H2052, NCI-H2452, and MSTO-211H cells untreated and treated with naftopidil analogues at concentrations as indicated for 24 h. In the graphs, each point represents the mean (± SEM) percentage of control (MTT intensities for cells untreated with naftopidil analogues) (*n* = 4 independent experiments).

**Figure 3:**
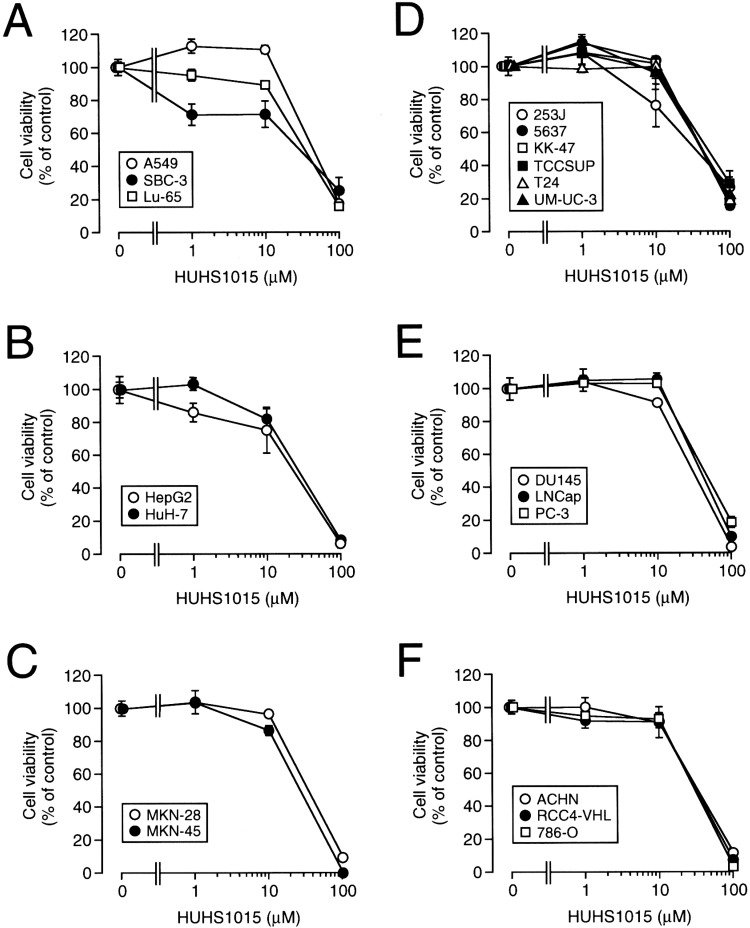
The effect of HUHS1015 on cell viability for a variety of human cancer cell lines. MTT assay was carried out in lung cancer cell lines (**A**); hepatoma cell lines (**B**); gastric cancer cell lines (**C**); bladder cancer cell lines (**D**); prostate cancer cell lines (**E**); and renal cancer cell lines (**F**), untreated and treated with HUHS1015 at concentrations as indicated for 24 h. In the graphs, each point represents the mean (±SEM) percentage of control (MTT intensities for cells untreated with HUHS1015) (*n* = 4 independent experiments).

We probed the anticancer effect of the naftopidil analogues using human MPM cell lines such as NCI-H28, NCI-H2052, NCI-H2452, and MSTO-211H cells. Of 21 analogues, the most beneficial effect was obtained with HUHS1015 (Figure 2) [9]. HUHS1015 also induced cell death in lung cancer cell lines A549, SBC-3, and Lu-65, hepatoma cell lines HepG2 and HuH-7, gastric cancer cell lines MKN-28 and MKN-45, bladder cancer cell lines 253J, 5637, KK-47, TCCSUP, T24, and UM-UC-3, prostate cancer cell lines DU145, LNCap, and PC-3, renal cancer cell lines ACHN, RCC4-VHL, and 786-O (Figure 3) [9], and colorectal cancer cell lines Caco-2 and CW2. This raises the possibility that HUHS1015 could be developed as an efficient anticancer drug. 

## 4. HUHS1015 Induces Caspase-Dependent and -Independent Apoptosis of Cancer Cells

In the analysis of terminal deoxynucleotidyl transferase-mediated dUTP nick end labeling (TUNEL) staining, HUHS1015 markedly increased TUNEL-positive cells after a 12-h treatment for NCI-H28, NCI-H2052, NCI-H2452, and MSTO-211H MPM cells, the extent reaching 61%, 69%, 37%, and 58% relative to total cells [9]. This indicates that HUHS1015 induces apoptosis of MPM cells.

In the enzymatic assay of caspase activities, HUHS1015 significantly activated caspase-3 for NCI-H28, NCI-H2052, NCI-H2452, and MSTO-211H MPM cells, but caspase-8 and -9 were not activated [9]. This suggests that HUHS1015-induced caspase-3 activation is not mediated through death receptors relevant to caspase-8 activation or mitochondrial damage relevant to caspase-9 activation. Amazingly, HUHS1015 upregulated expression of mRNAs for Bax, Puma, Hrk, and Noxa in NCI-H2052 cells and for Puma, Hrk, and Noxa in MSTO-211H cells [10]. The Bcl-2 family is further divided into three classes: (i) the Bcl-2 subfamily, which includes Bcl-2, Bcl-X_L_, Bcl-w, Mcl-1, and A1; (ii) the Bax subfamily, which includes Bax, Bak, and Bok; and (iii) the BH3-only Bcl-2 family, which includes Bad, Bik, Bid, Bim, Blk, Hrk, BNIP3, Puma, and Noxa [11,12]. The Bcl-2 subfamily serves as an anti-apoptotic factor. In contrast, the Bax subfamily and the BH3-only Bcl-2 family make pores in the mitochondria, allowing the release of apoptosis-related factors such as cytochrome c, apoptosis-inducing factor (AIF), AIF-like mitochondrion-associated inducer of death (AMID), and Smac/DIABLO, leading to caspase-9-dependent and independent apoptosis. HUHS1015, in the light of these facts, may induce apoptosis of MPM cells through a mitochondria-mediated and caspase-independent pathway.

**Figure 4:**
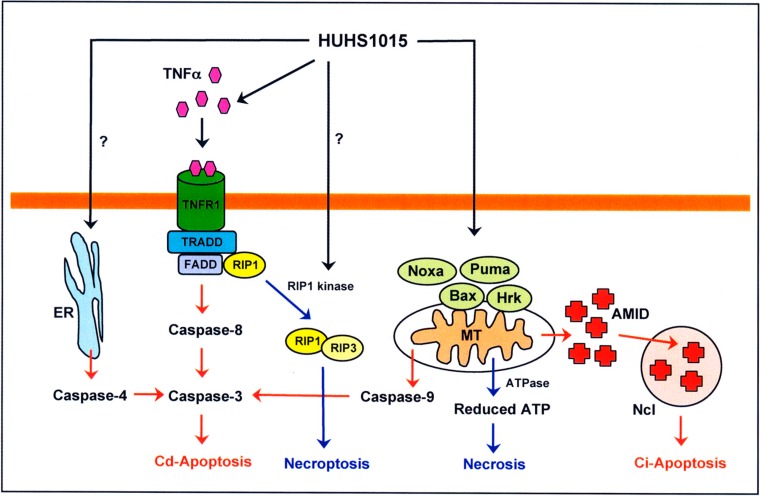
Schematic pathways for HUHS1015-induced cell death. Cd-Apoptosis: caspase-dependent apoptosis; Ci-Apoptosis: caspase-independent apoptosis; ER: endoplasmic reticulum; MT: mitochondria; Ncl: nucleus.

Intriguingly, HUHS1015 still activated caspase-4 for all the investigated MPM cell lines (Figure 4) [9]. Moreover, HUHS1015 activated caspase-3, -4, and -8 in MKN45 poorly differentiated gastric cancer cells, although no activation of caspase-3, -4, -8, and -9 was found in MKN28 well differentiated gastric cancer cells. HUHS1015 also activated caspase-3 and -9 in Caco-2 colorectal cancer cells and caspase-3, -4, -8, and -9, with huge activation of caspase-3 in CW2 colorectal cancer cells. Caspase-4 is activated in association with endoplasmic reticulum (ER) stress [13,14,15]. It is presently unknown whether HUHS1015 induces ER stress and how HUHS1015 activates caspase-4. Overall, HUHS1015 induces caspase-dependent and -independent apoptosis of cancer cells, depending upon cancer cell types.

## 5. HUHS1015 Activates Caspase-8 by Upregulating Tumor Necrosis Factor α (TNFα)

HUHS1015 upregulated expression of the mRNAs for FasL, Fas, Fas-associated death domain (FADD), TNFα, TNF receptor 1 (TNFR1), and TNFR1-associated death domain protein (TRADD) in MKN45 cells. In addition, HUHS1015 significantly increased expression of TNFα protein in MKN45 cells, although expression of proteins for FasL, Fas, FADD, TNFR1, and TRADD was not increased.

Caspase-8 is activated through death receptors. The death receptor Fas, activated by FasL, recruits the adaptor protein FADD to aggregate procaspase-8, which cleaves to one other to initiate an active form of caspase-8 and, in turn, to activate the effector caspase-3 [16]. TNFα activates TNFR1, which forms a complex of TRADD/FADD/procaspase-8 to activate caspase-8 followed by the effector caspase-3 [17]. Collectively, HUHS1015 could activate caspase-8 by activating TNFR1 in association with upregulation of TNFα expression, followed by activation of the effector caspase-3, to induce apoptosis.

## 6. HUHS1015 Induces Caspase-Independent Apoptosis by Accumulating AMID in the Nucleus

HUHS1015 significantly increased nuclear localization of AMID in parallel with decreased cytosolic localization in MKN28 cells [18]. An increase in the nuclear localization of AIF was not obtained with HUHS1015, but, conversely, nuclear localization of AIF was decreased [18]. HUHS1015 had no effect on expression of mRNAs and proteins for AIF and AMID in MKN28 cells [18].

In response to lethal signals, AIF is translocated from the mitochondria into the nucleus, where it binds to the nuclear DNA, thereby causing chromosomal condensation, margination, and large-scale DNA fragmentation (approximately 50-kb fragments) [19,20]. The AIF homologue AMID is identified as a human pro-apoptotic protein and designated as p53-responsive gene 3 [21,22,23,24,25,26]. AMID is preferentially localized in the outer mitochondrial membrane or the cytoplasm. In response to apoptotic stimuli, AMID is translocated in the nucleus and non-specifically binds to DNAs to induce DNA fragmentation, *i.e.*, apoptosis [23,24,25,26]. Taken together, HUHS1015 induces caspase-independent apoptosis by accumulating AMID in the nucleus.

## 7. HUHS1015 Induces Necrosis (Necroptosis) of Cancer Cells

In the flow cytometry using propidium iodide (PI) and annexin V, PI is a marker of dead cells and annexin V, which detects externalized phosphatidylserine residues, is a marker of apoptotic cells [27]. In this assay, each population of PI-positive/annexin V-negative, PI-negative/annexin V-positive, or PI-positive/annexin V-positive cells corresponds to primary necrosis, early apoptosis, and late apoptosis/secondary necrosis, respectively [28]. 

HUHS1015 increased the populations of PI-positive/annexin V-negative, PI-negative/annexin V-positive, and PI-positive/annexin V-positive cells in NCI-H2052 and MSTO-211H cell lines [10]. HUHS1015, alternatively, increased the populations of PI-positive/annexin V-negative and PI-positive/annexin V-positive cells in MKN28 [18], Caco-2, and CW2 cell lines. These data indicate that HUHS1015 induces both necrosis and apoptosis of a variety of cancer cells. Notably, HUHS1015 damaged mitochondrial membrane potentials in these cells. HUHS1015-induced cell death was prevented by necrostatin-1 (Nec-1), an inhibitor of necroptosis (an inhibitor of receptor interacting protein 1 (RIP1) kinase) [18]. Consequently, HUHS1015 appears to induce necrosis (necroptosis) of cancer cells as well.

Cell death is classified into three types: apoptosis, necrosis, and necroptosis. Necroptosis is regarded as an alternative form of programmed cell death [29]. Death receptors such as TNFα receptor and Fas or pro-apoptotic Bcl-2 family members related to mitochondrial damage mediate both in apoptosis and necroptosis. RIP1 associated with death receptors forms complex IIa including FADD and caspase-8, causing activation of caspase-8 followed by caspase-3 [30]. In a different pathway, caspase-8 proteolyzes Bid into truncated Bid (tBid), which makes pores in the mitochondria, allowing release of apoptosis-related factors involving activation of caspase-9 and the effector caspase-3 [31]. RIP1 is phosphorylated by RIP1 kinase and forms complex IIb, together with RIP3, to induce necroptosis [30]. Apoptotic stimuli such as oxidative stress cause pro-apoptotic Bcl-2 family member-mediated damage of mitochondria, thereby releasing cytochrome c to activate caspase-3/9 and induce mitochondrial apoptosis [32]. Mitochondrial damage, alternatively, activates ATPase and reduces intracellular ATP concentrations to induce necroptosis [33]. Overall, HUHS1015 is likely to induce both necrosis (necroptosis) and apoptosis of cancer cells.

## 8. HUHS1015 Suppresses Tumor Growth in Xenograft Model Mice

HUHS1015 clearly suppressed tumor growth in mice inoculated with NCI-H2052 cells, with a potential much greater than that for paclitaxel [10]. The survival rate at two months after HUHS1015 treatment was 100%, but it was only 30% for paclitaxel-treated mice [10]. In mice inoculated with MKN45 cells, HUHS1015 obviously suppressed tumor growth, but naftopidil otherwise had no significant effect. This indicates that the naftopidil analogue HUHS1015 is more effective on gastric cancer cells than naftopidil by itself. The anticancer drugs cisplatin, paclitaxel, and irinotecan also suppressed tumor growth, and the order of the potential for all the investigated compounds was irinotecan >> HUHS1015≒ cisplatin > paclitaxel > naftopidil. The survival rate at 33 days’ treatment with HUHS1015 was 100%, while that for cisplatin, irinotecan, and paclitaxel treatment was 43%, 71%, and 86%, respectively. HUHS1015 also inhibited tumor growth in mice inoculated with CW2 cells, with the survival rate being 100% at 33 days’ treatment. In addition, no remarkable body weight loss was found throughout experiments and the blood examination exhibited no liver and renal dysfunction. Overall, these data show that HUHS1015 serves as a beneficial anticancer drug, with lesser side effects.

## 9. Conclusions

The naftopidil analogue HUHS1015 induces both necrosis (necroptosis) and apoptosis in a wide variety of cancer cells. The former may be caused by intracellular ATP reduction in association with mitochondrial damage or activation of the RIP1 kinase (Figure 4). The latter may be due to caspase activation through the mitochondria and TNFα receptor for caspase-dependent apoptosis and to AMID accumulation in the nucleus for caspase-independent apoptosis (Figure 4). HUHS1015 effectively prevented tumor growth in the xenograft model mice, with lesser side effects. HUHS1015 could, thus, be developed as a new type of promising anticancer drug.

## Abbreviation

HUHS1015: 1-[2-(2-methoxyphenylamino)ethylamino]- 3-(naphthalene-1-yloxy)propan-2-ol
MPM: malignant pleural mesothelioma
PKC: protein kinase C
TUNEL: terminal deoxynucleotidyl transferase-mediated dUTP nick end labeling
AIF: apoptosis-inducing factor
AMID@: AIF-like mitochondrion-associated inducer of death
ER: endoplasmic reticulum
TNFα: tumor necrosis factor α
FADD: Fas-associated death domain
TNFR1: TNF receptor 1
TRADD: TNFR1-associated death domain protein
PI: propidium iodide
Nec-1: necrostatin-1
RIP1: receptor interacting protein 1
tBid: truncated Bid
